# Managing Different Spectrums of Tuberous Sclerosis Facial Angiofibroma: A Report of Two Cases

**DOI:** 10.7759/cureus.35200

**Published:** 2023-02-19

**Authors:** Khairul Mustaqim, Ahmad Faiz Najmuddin Mohd Ghazi, Shawaltul Akhma Harun Nor Rashid, Syafiq Mohamad

**Affiliations:** 1 Surgery, Universiti Sains Malaysia School of Medical Sciences, Kota Bharu, MYS; 2 Plastic and Reconstructive Surgery, Hospital Raja Perempuan Zainab II, Kota Bharu, MYS

**Keywords:** multisystem hamartomas, electrosurgery, serial excision, facial angiofibroma, tuberous sclerosis

## Abstract

Tuberous sclerosis is one of the rarest genetically linked disorders that can affect a multitude of body systems in various forms. Patients with facial angiofibroma may face issues arising from the various modalities and approaches that can be applied. Moreover, surgeons also face challenges in preparing patients for specific interventions. Here, we are reporting the two spectrums of this condition that may present, along with how we managed patients in our center. The first case is a severe form of tuberous sclerosis involving the skin as well as neurological manifestation, while the second case is a milder form. Both were treated with serial excision and electrosurgery, respectively. Facial angiofibroma in tuberous sclerosis can present in various spectrums. Serial excision and electrosurgery are deemed acceptable. Few advancements have been made in the management of this condition, and combination therapies have shown favorable outcomes. Optimizing patient comorbidities is imperative before intervention and multidisciplinary team involvement would ensure patient safety.

## Introduction

Tuberous sclerosis (TS) or tuberous sclerosis complex or Bourneville disease is one of the rarest genetically linked disorders that can affect a multitude of body systems in various forms.

This condition is usually described as multisystem hamartomas inclusive of the kidneys, brain, skin, heart, and lungs; hence, the presentation can vary depending on the specific system involved.

Occurring in one out of 6,000 to 10,000 individuals, this autosomal dominant condition may exhibit dermatological condition which ranges from hypomelanotic macules (ash leaf spots) to facial angiofibroma (FA) [1].

Patients of TS with facial angiofibroma undergoing plastic surgery may encounter difficulties stemming from various methods and approaches available, along with the challenges faced by surgeons in preparing the patients for specific procedures.

Here, we are reporting the two spectrums of this condition which may present, as well as how we managed patients in our center.

## Case presentation

Patient one

A 21-year-old gentleman, with a known case of TS, presented with extensive facial lumps since the age of seven years. The lumps increased in number and size over the years in association with bleeding on scratching. The patient had a couple of hospitalizations for blood transfusion for microcytic hypochromic anemia due to bleeding from the facial lumps.

The patient was also under follow-up for epilepsy, intellectual disability, and autism. He was bedridden and unable to communicate verbally since childhood. Raised by a single mother with low socioeconomic background, maintaining proper follow-up and optimization of his condition was challenging. The patient also suffered from other comorbidities along with his neurological and dermatological issues.

**Figure 1:**
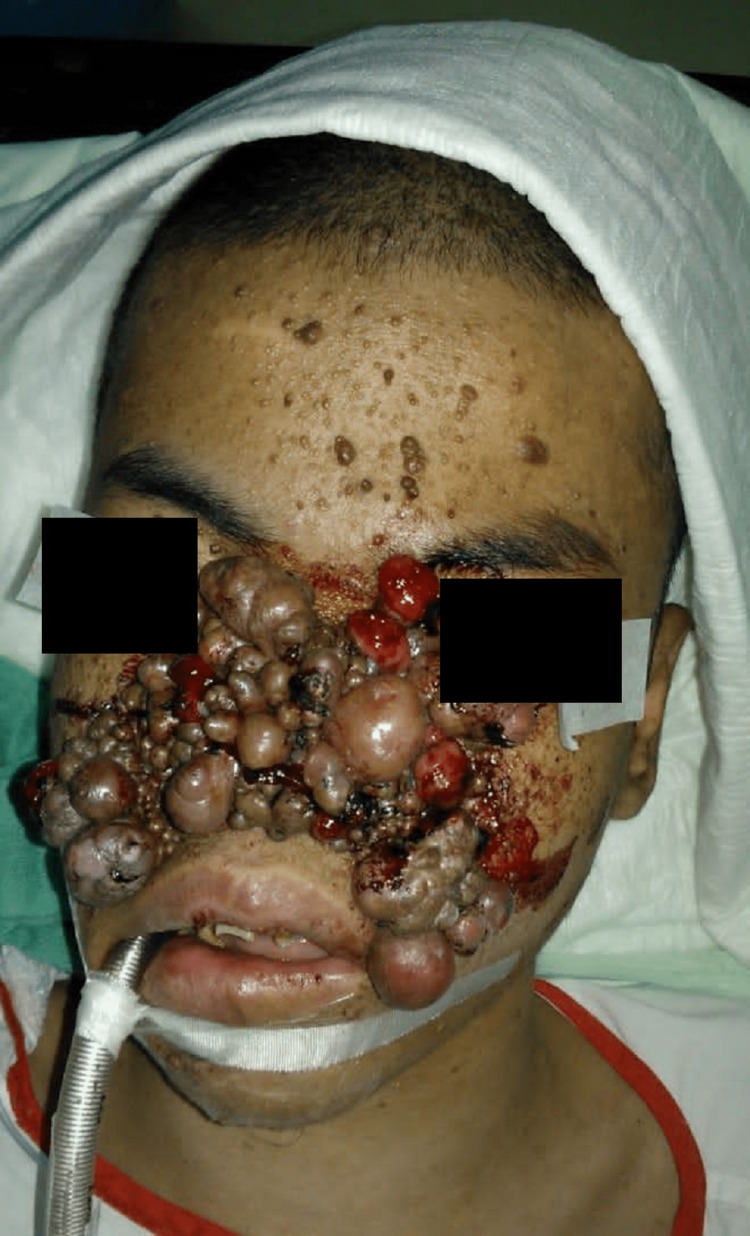
Preoperative image depicting the severe facial angiofibroma with multiple bleeding lumps centrally.

**Figure 2:**
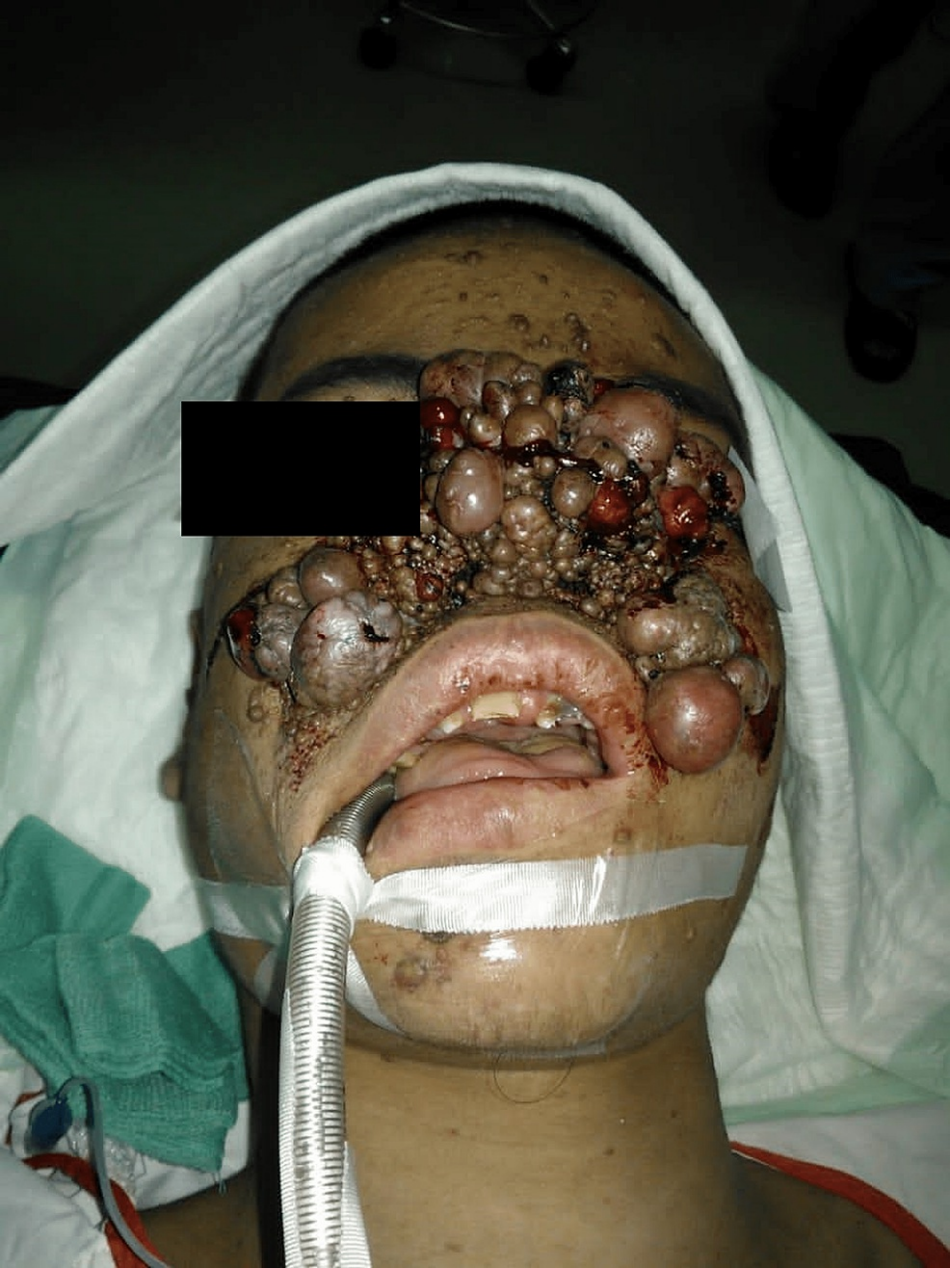
Intraoperative image, worm-eye view highlighting the severity of the facial angiofibroma blocking the patient’s nostril and obstructing his nasal breathing.

Clinically, there were extensive, multiple-sized lumps on the face, more at the center covering his nose including the nares with contact bleeding, as seen in Figure 1 and Figure 2.

The patient also exhibited hypomelanotic macules all over the body and facial areas.

After consulting neurology, anesthesia, and internal medicine teams via a multidisciplinary meeting, we subjected the patient to serial electrosurgery excision under general anesthesia to remove the bleeding facial lumps. The operations were uneventful and the patient was discharged within three days after each surgery. The histopathological examinations were positive for angiofibroma.

**Figure 3:**
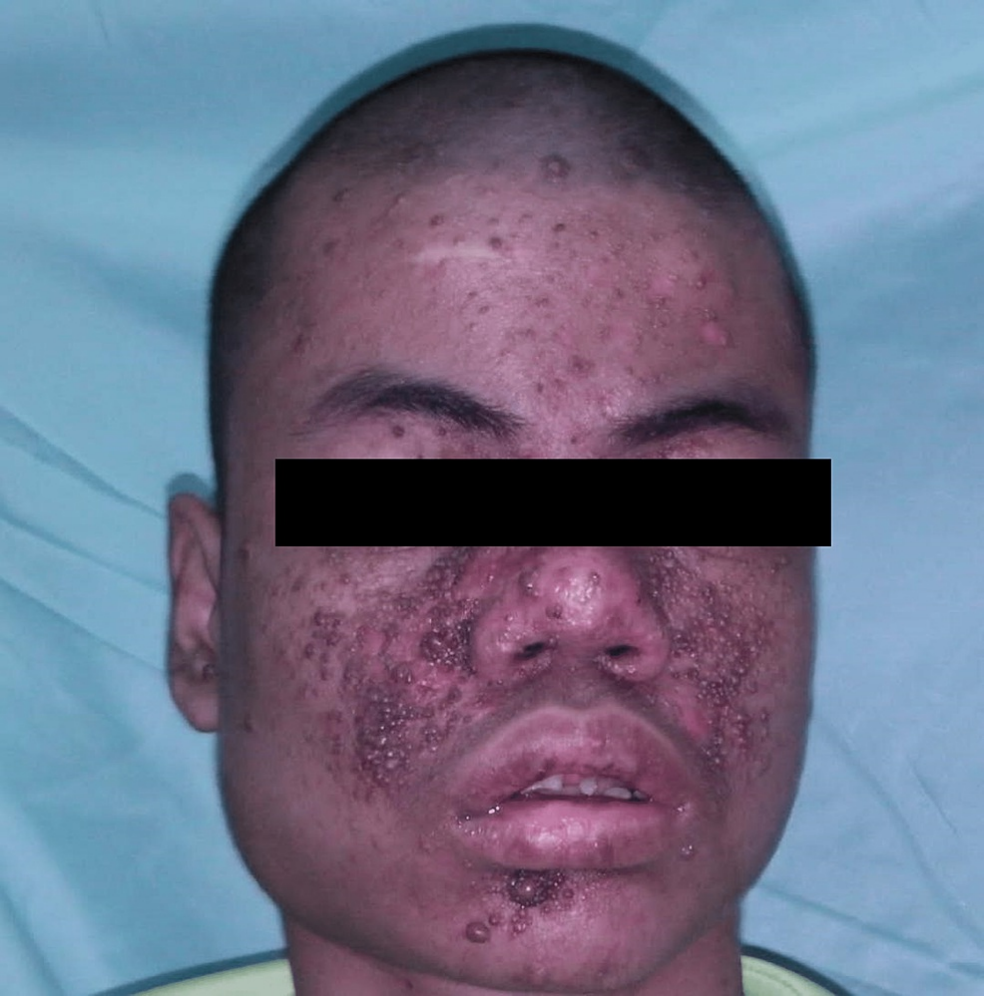
Postoperative image after multisession excisions.

This is a severe form of TS with mental retardation and epilepsy exhibiting extensive FA with contact bleeding causing anemia. After the multisession excision of the FA, the patient has improved cosmesis (as shown in Figure 3) and no more hospitalizations for anemia.

Patient two

A healthy 16-year-old boy with facial skin lesions, especially over the nasal area, was initially under dermatology follow-up for seborrheic hyperplasia and tinea cruris. The patient was referred to the plastic surgery team after he started to exhibit progressive discomfort and some visual disturbances from the lesion under the right eyelid.

**Figure 4:**
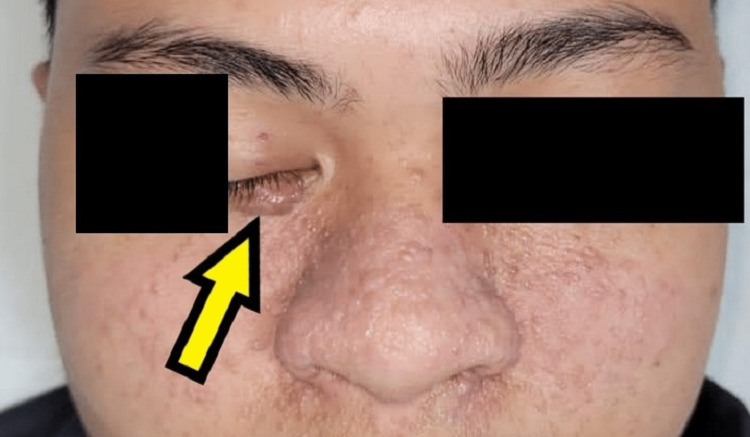
Preoperative image of the bothersome facial angiofibroma lesion over the right lower eyelid (yellow arrow).

Clinically, there was a 1.2 × 0.5 cm papule over the right lower eyelid (as shown in Figure 4), and soft small papules over the nose and cheeks.

**Figure 5:**
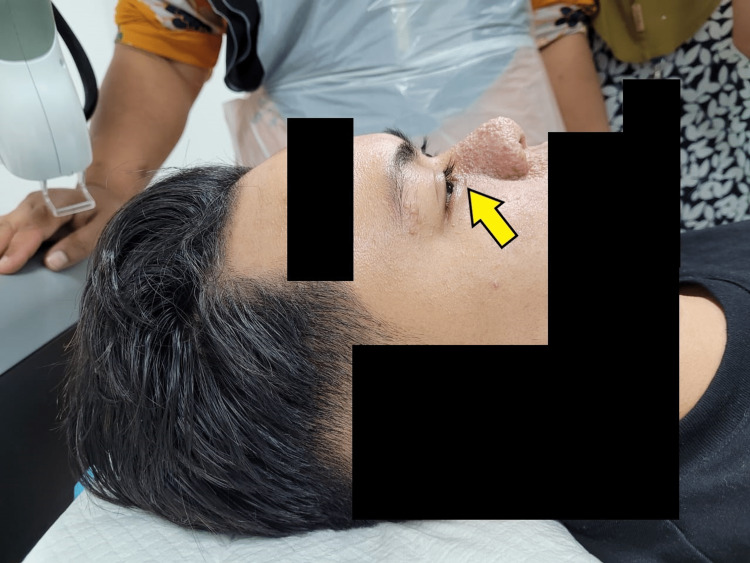
Post-intervention image after electrosurgery excision (yellow arrow).

The patient was subjected to serial conventional CO_2_ laser ablation under topical anesthesia (EMLA cream) over the nose and bilateral cheeks. We used a single pass of the conventional laser set at 18 mJ^ ^and a density of 0.5. Ultimately, the patient underwent electrosurgery excision over the right lower eyelid in view of its progressive nature and some visual impairment. The histopathology examinations confirmed the diagnosis of angiofibroma. This is a milder form of TS with electrosurgery excision that improved the patient’s visual comfort and cosmesis (as shown in Figure 5).

## Discussion

TS can be diagnosed based on the clinical presentation of patients either fulfilling the major or minor criteria [2] depending on the organs involved.

The main concern in patients with TS is mostly FA, affecting up to 75-80% of the patients [3]. Interventions are offered to those with a debilitating form causing bleeding or infection, as well as to those with emotional distress due to cosmetic disfigurement [4].

Depending on the extent of dermatological presentation, some of the modalities used in managing these angiofibromas include cryosurgeries, desiccation and curettage, dermabrasion, limited excision, shaving, CO_2_, laser and argon laser [5]. The intervention of choice comes with varying success rates and differs from centers. Topical medical therapies can also be an alternative in mild forms of FA or in younger patients.

In our case, serial excision was preferred due to the severe form of FA affecting patients’ vision. Excision of these angiofibromas may benefit patients in terms of the overall outcome in severe form with a few drawbacks. The surgical excision may pose a challenge, especially in patients who require general anesthesia in the background of neurological or cardiovascular involvement of TS. Perioperative optimization is imperative. Complications associated with the excision of FA may include a hypertrophic scar, pigmentation disorders, pain, and infections, as well as the failure to tackle the issue in a single setting.

Electrosurgery, on the other hand, is the application of a diathermic apparatus that delivers precise energy to targeted tissue aimed for moderate-to-severe FA [2]. Multiple advancements have been made to concentrate the energy transfer without damaging the surrounding tissues causing scarring and pigmentations.

A combination of treatment modalities is applied to improve outcomes as well as reduce complications in most situations.

Another consideration in treating TS patients is the optimization of patients’ comorbidities associated with heterogeneous TS involvement in other systems. Neurological manifestation should be dealt with by addressing issues such as mental retardation and seizure control. Symptomatic cardiovascular and renal manifestations should also be addressed based on organ-specific management. A multidisciplinary team approach should be undertaken to achieve a successful and safe intervention in TS patients [1].

## Conclusions

FA in TS can present in various spectrums. Serial excision and electrosurgery are deemed acceptable following the standard for treating TS. Advancements have been made and combination therapies can help achieve favorable outcomes. Optimizing patient comorbidities is imperative prior to intervention, and multidisciplinary team involvement can help ensure patient safety.
